# The unmet information needs, quality of life, and care experiences of patients with neuroendocrine tumours (NETs) at follow-up: 6 months from diagnosis

**DOI:** 10.1007/s00520-023-08034-5

**Published:** 2023-09-15

**Authors:** Lisa Guccione, Karla Gough, Allison Drosdowsky, Timothy Price, Nick Pavlakis, David Wyld, David Ransom, Michael Michael, Penelope Schofield

**Affiliations:** 1 Department of Health Services Research, Peter MacCallum Cancer Centre, Melbourne, VIC Australia; 2 Sir Peter MacCallum Department of Oncology, Faculty of Medicine, Dentistry and Health Sciences, The University of Melbourne, Melbourne, Australia; 3 Haematology and Oncology, The Queen Elizabeth Hospital, Woodville South, South Australia Australia; 4 Department of Medical Oncology, Royal North Shore Hospital, St Leonards, New South Wales Australia; 5 Department of Medical Oncology, Royal Brisbane and Women’s Hospital, Brisbane, QLD Australia; 6 Faculty of Medicine, University of Queensland, Brisbane, Australia; 7 Medical Oncology, Fiona Stanley Hospital, Murdoch, WA Australia; 8 Department of Medical Oncology, Peter MacCallum Cancer Centre, Melbourne, Australia; 9 Upper Gastrointestinal Cancer Service, Peter MacCallum Cancer Centre, Melbourne, Australia; 10 Department of Psychology and Iverson Health Innovation Research Institute, Swinburne University, Melbourne, Australia

**Keywords:** Healthcare preferences, Mixed-methods multi-site study, Supportive care services, Patients

## Abstract

**Objectives:**

To identify changes in the healthcare preferences, patient experiences, and quality of life of patients with NETs at 6-month follow-up, informing the design of supportive care services.

**Methods:**

This study presents 6-month follow-up data of a mixed-methods multi-site study. Demographic, clinical, and patient-reported outcome questionnaire data was collected.

**Results:**

High percentages of suboptimal experiences of care were reported. Patients reported less positive experiences with being involved in decisions about their care and treatment; their family or someone close to them having the opportunity to talk to their cancer doctor, or having their family or someone close to them receive all the information they need to help care for them at home. Patients also reported negative experiences for on the information about their cancer accessible online and the usefulness of the information they accessed. Differences between baseline and follow-up scores were mostly not significant apart from anxiety and sleep disturbance scales,

**Conclusions:**

Patients with NETs report difficulties in accessing and understanding written information that is persistent over time.

**Practice implications:**

Outcomes will inform the design and development of an informational resource aimed at facilitating improved understanding for patients with NETs.

## Background

Considered to be rare, neuroendocrine tumours (NETs) are a heterogeneous group of malignancies that are becoming more prevalent worldwide [1, 2]. Within the USA, population-based retrospective cohort studies have found incidence rates have increased 6.4 fold over the past five decades [3]; and similarly in Canada, incidence rates had increased from 2.48 to 5.86 per 100,000 per year [4]. Prognostic factors, such as primary site of the NET, tumour stage, and pathological grade, have been reported to predict survival outcomes for people with NETs [5, 6]. Although most commonly found in gastrointestinal and bronchopulmonary sites [1, 7, 8], NETs can originate almost anywhere in the body, and therefore survival rates can also vary [6, 9]. The severity of symptoms associated with NETs and symptom burden can also differ between patients. Depending on the secretory potential of the tumour, tumours that secrete hormones and are functional in nature can lead to clinical syndromes that produce highly distressing symptoms for patients. For others, the presentation of NETs that are non-functional can be indolent and asymptomatic in nature even with widespread disease [10, 11]. Like many other rare cancers, due to their non-specific presentation and the lack of awareness within the community and in clinical settings [12], NETs are poorly understood.

Navigating through a diagnosis of NETs presents unique challenges for both patients and clinicians. First, attaining an accurate diagnosis is often delayed due to the nature of symptoms patients present with; commonly misdiagnosed with conditions such as irritable bowel syndrome or dyspepsia [13–15]. Patients report difficulties in navigating through unclear treatment pathways and disease management with no disease-specific supportive care available that addresses the specific needs of the NET patient population [16]. Low levels of satisfaction with the organisation of care have also been reported and are often associated with impaired psychosocial function and increased anxiety [17].

The priority of needs reported by the NET patient population has predominantly been within psychological, physical, and daily living, and health system information domains, with information needs being most prevalent for patients diagnosed within the previous 6 months [18, 19]. However, despite the prominent need for disease-related information, it has been previously found that 69% of newly diagnosed patients reported either not receiving any written information about their cancer, or when it was received, not understanding it. Most patients access disease-related information online [20]; nevertheless, two-thirds of newly diagnosed NET patients do not find the online information resources useful [19].

Information needs are known to be greatest at diagnosis and at the start of treatment, declining over time [21]. We have previously reported that, at diagnosis, patients with both functioning and non-functioning NETs report disease-specific information to be of a high priority [19]. Specifically, most patients reported negative experiences with understanding the explanation of what was wrong with them (67%), receiving written information about their cancer (69%), their family/carer receiving all the information required to care for them (61%), and the usefulness of information about NETs online (66%). Most reported at least one moderate-to-high need for disease-specific information (63%) [19].

The aim of this study was to identify any changes in the care preferences, patient experiences, and quality of life (QoL) of patients with NETs at 6-month follow-up. We sought to understand if anxiety, depression, fatigue, pain interference, pain intensity, sleep disturbance, physical function, satisfaction with social roles and activities, supportive care needs, and experiences of the health care system, as well as general and disease-specific health-related QoL and clinical characteristics, changed over time.

## Methods

### Study design and setting

This study constitutes an analysis of data from phase one of a three-phase mixed methods study as outlined in the published protocol [22]. Quantitative data was collected at baseline (within 6 months from diagnosis) and then 6 months later. Ethics approval was obtained for this study from the Human Research Ethics Committee (HREC) of Peter MacCallum Cancer Centre (16/08L) and other participating sites (Royal North Shore Hospital and Northern Cancer Institute, NSW; the Lyell McEwin Hospital, SA; Royal Brisbane Hospital, QLD; and the Fiona Stanley Hospital, WA).

### Eligibility and recruitment

Eligibility criteria were as follows:

1. A histologically confirmed diagnosis of a NET within the past 6 months
2. A NET classified as either functioning or non-functioning by their treating oncologist
3. Aged 18 years of age or older
4. Able to speak and read English
5. Well enough to participate in the study as determined by the patient’s treatment team, and no psychological or cognitive difficulties that would preclude study participation as determined by the patient’s treatment team based on a cognitive and/or psychiatric assessment or the patient’s disclosed medical history.

Patients were withdrawn if their cancer could no longer be detected or if they withdrew consent. Histological confirmation was derived from pathology reports from tumour biopsies using the World Health Organisation (WHO) classification [23]. These sites were documented. Hereditary syndromes such as multiple endocrine neoplasia (MEN 1) were not recorded.

For each site, a researcher screened and identified potentially eligible patients. Eligibility was then confirmed by the treating clinician prior to any approach, clarifying details from the medical records. Treating clinicians also classified the patient’s tumour as either functional or non-functional based on their clinical presentation with regard to hormonal hypersecretory syndrome. The study was then described to patients, and they were provided with a copy of the Participant Information and Consent Form (PICF), a baseline questionnaire and a reply-paid envelope. Patients were given an opportunity to ask questions and were informed that their involvement in the study was completely voluntary. At the time of consent, patients were asked to sign the PICF and complete the baseline questionnaire. Patients who declined to participate were asked to provide consent for the collection of basic demographic and clinical information to support an assessment of recruitment bias. Reasons for refusal were recorded. Profiles of the psychosocial, QoL, and clinical characteristics of patients with functioning, and non-functioning NETs were then developed based on patient-reported outcome data and clinical data extracted from the electronic medical record.

### Measures

Demographic and clinical information was collected from participant’s medical records. Patient-reported outcomes are listed below and described in more detail in the published protocol [22].

Patient-Reported Outcomes Measurement Information System (PROMIS®) short forms consisted of emotional distress and anxiety (short form 7a), depression (short form 8b), fatigue (short form 7a), pain interference (short form 6b), pain intensity (short form 3a), sleep disturbance (short form 8b), physical function (short form 10a), and satisfaction with social roles and activities (short form 6a); the Supportive Care Needs Survey (SCNS), a 34-item questionnaire assessing needs scales across physical and daily living, psychological, sexuality, patient care and support, and health system and information; the European Organisation for Research and Treatment of Cancer Quality of Life Questionnaire (EORTC QLQ-C30), a 30-item questionnaire consisting of global health status scale, five functional scales, three symptom scales and six single items assessing dyspnoea, sleep disturbance, appetite loss, constipation, diarrhoea, and financial impact [24]; the European Organisation for Research and Treatment of Cancer Quality of Life Questionnaire—Neuroendocrine Carcinoid Module (QLQ-GINET21), a 21-item questionnaire incorporating five scales (endocrine, G.I, treatment, social function and disease related worries scale) and 4-single items (muscle/bone pain symptom, sexual function, information/communication function and body image), and a subset of 21 items from the National Cancer Patients Experience Survey about their experiences of the health care system following their diagnosis.

### Sample size

The sample size was pragmatic based on study timeframes and resources. Over 32 months, a total number of 1155 patients were screened, 975 were ineligible (*n* = 932 diagnosed > 6 months, *n* = 25 resected before baseline, *n* = 9 did not read/write in English, *n* = 9 missing). A further 22 patients were not approached due to reasons such as no follow-up appointment, clinician discretion, and patient too unwell, and 22 patients declined to participate. The remaining 138 consented to the study. At the follow-up data collection timepoint, 100 participants had completed patient reported outcome measures (PROMs) at follow-up assessment.

### Statistical methods

Scoring and analysis was undertaken in SPSS Version 25 (Chicago IL, USA). Patient-reported outcome measures were scored in accordance with developer/author guidelines. Responses to SCNS-SF items were recoded to a set of discrete variables comprising two categories: no/low need and moderate/high need. Responses to CPES items were also recoded to a set of discrete variables comprising two categories: optimal (*the most positive response*) and sub-optimal (*less positive responses*) experiences of care. Responses that indicated *don’t know/can’t remember* or *no need for information/support* were excluded. CPES items with Likert-type responses were not recoded.

Descriptive statistics were used to summarize sample characteristics, and responses to patient-reported outcome measures at baseline and follow-up, including recoded responses. Paired samples *t*-tests were used to compare responses to PROMIS and EORTC scales/items at baseline and follow-up. Kazis effect size (ES) estimates were used to characterise the sizes of observed differences [25]. Individual change scores on PROMIS and EORTC scales/items were recoded to a set of discrete variables comprising five categories: (1) improved by 5–9, (2) improved by at least 10 points, (3) remained stable (improved or deteriorated by less than 5 points), (3) deteriorated by 5–9 or (5) deteriorated by at least 10 points, then valid percentages calculated. These scores reflect changes in patient-reported outcomes that present clinically meaningful differences [26, 27].

## Results

### Study profile

**Fig. 1:**
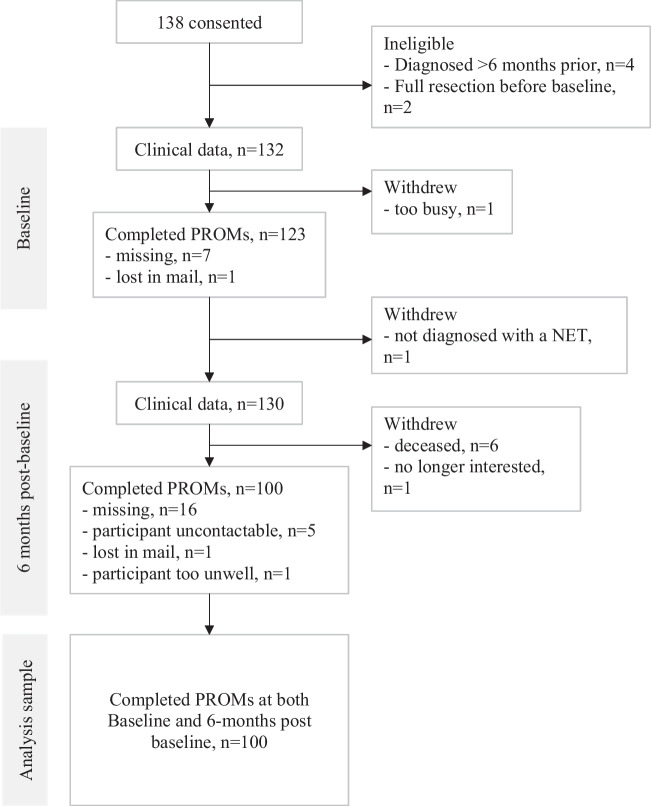
Participant flow diagram

The flow of participants through the study is presented in Fig. 1. In total, 100 of 123 participants with baseline PROMs data completed the follow-up assessment. The sociodemographic and clinical characteristics of these participants are summarised in Table 1. As previously described Guccione et al. (2021), differences between the experiences of patients with functioning and non-functioning NETs were not statistically significant, and therefore, results have not been stratified by NET sub-group.

**Table 1:** Participant characteristics (*n* = 100)^a^

Characteristic	*n*	Valid %
Age		
Mean (SD)	61.8 (11.7)	
Range	28, 91	
Sex		
Male	49	49.0
Female	51	51.0
Marital status		
Single	11	11.2
Married/de facto	71	72.4
Separated/divorced	11	11.2
Widowed	5	5.1
Employment status		
Employed	31	31.3
Not employed	68	68.7
Highest level of education		
Primary schooling	2	2.0
Secondary schooling	36	36.7
Tertiary schooling	41	41.8
Trade/TAFE college	19	19.4
Living arrangements		
With spouse/partner	49	49.5
With spouse/partner and children	22	22.2
By yourself/independently	16	16.2
With children only	6	6.1
With other family member/s	4	4.0
With non-family members	1	1.0
Other	1	1.0
Country of birth		
Australia	80	81.6
Not Australia	18	18.4
English as first language		
Yes	95	96.0
No	4	4.0
Site of disease		
Pancreatic	37	37.4
Small bowel	36	36.4
Lung	12	12.1
Cancer of unknown primary	5	5.1
Liver	3	3.0
Other	6	6.1
Grade of disease (baseline)		
1	32	34.8
2	48	52.2
3	12	13.0
Stage of disease (baseline)		
1	0	0.0
2	1	2.2
3	5	11.1
4	39	86.7
At 6-month follow-up, cancer status		
No change	56	56.0
Progressed	20	20.0
Regressed	23	23.0

^a^Missing/not recorded: marital status, *n* = 2; employment status, *n* = 1; highest level of education, *n* = 2; living arrangements, *n* = 1; country of birth, *n* = 2; English, *n* = 1; site of disease, *n* = 1; grade of disease, *n* = 8; stage of disease, *n* = 55; grade or stage change at 6-month follow-up, *n* = 20

### Patient-reported outcomes

Descriptive statistics for responses to the PROMIS and EORTC scales/items are provided in Table 2, as are the proportions of participants whose scores improved (5–9 or > 10 points), remained stable, or deteriorated (5–9 or > 10 points) between baseline and follow-up.

**Table 2:** Descriptive and change statistics for PROMIS short forms and EORTC measures (*n* = 100)

Measure/scale or item^a^	*n*	Baseline	Follow-up	M diff (95% CI)	Effect size^b^	Within individual changes^c^				
						%Improved		%Stable	%Deteriorated	
		M (SD)	M (SD)			10 pts	5 pts	< 5 pts	5 pts	10 pts
PROMIS										
Anxiety	100	54.6 (10.2)	51.5 (10.5)	− 3.1 (− 4.7, − 1.4)	0.30	21	15	54	4	6
Depression	100	50.6 (10.0)	48.7 (10.5)	− 1.9 (− 3.8, − 0.1)	0.19	21	7	54	11	7
Fatigue	100	52.0 (8.9)	53.3 (7.9)	1.3 (− 0.2, 2.8)	0.15	6	13	49	20	12
Pain interference	100	49.2 (9.1)	48.0 (8.6)	− 1.3 (− 2.5, 0.0)	0.14	10	12	66	6	6
Pain intensity	97	38.7 (9.0)	38.1 (8.8)	− 0.6 (− 2.2, 1.0)	0.07	8	10	63	11	7
Sleep disturbance	99	51.5 (9.7)	53.5 (9.4)	2.0 (0.2, 3.8)	0.20	5	16	45	16	17
Physical function	100	46.8 (10.3)	47.4 (8.0)	0.6 (− 1.1, 2.3)	0.06	4	16	64	8	8
Satisfaction with social roles and activities	100	52.1 (10.2)	51.7 (10.5)	− 0.4 (− 2.0, 1.2)	0.04	10	16	52	14	8
EORTC QLQ C30										
Functioning scales										
Global health status	96	68.8 (21.4)	70.3 (20.1)	1.5 (− 2.6, 5.6)	0.07	24	11	23	19	23
Physical functioning	98	84.1 (18.6)	84.0 (18.5)	0.0 (− 3.4, 3.3)	0.00	17	13	36	11	22
Role functioning	97	76.3 (29.6)	79.9 (26.5)	3.6 (− 2.1, 9.3)	0.12	30	0	48	0	22
Emotional functioning	98	75.7 (23.8)	75.9 (25.2)	0.2 (− 3.8, 4.3)	0.01	24	5	35	13	22
Cognitive functioning	98	80.1 (21.8)	79.3 (19.2)	− 0.9 (− 5.1, 3.4)	0.04	23	0	43	0	34
Social functioning	98	77.4 (27.6)	78.2 (24.2)	0.9 (− 4.1, 5.8)	0.03	24	0	52	0	23
Symptom scales										
Fatigue	98	34.0 (26.4)	36.3 (27.3)	2.4 (− 3.1, 7.9)	0.09	34	1	27	1	38
Nausea/vomiting	98	10.4 (17.2)	10.4 (15.4)	0.0 (− 4.0, 4.0)	0.00	17	0	62	0	20
Pain	98	20.7 (26.0)	19.4 (26.1)	− 1.4 (− 5.9, 3.1)	0.05	24	0	54	0	21
Dyspnoea	97	17.5 (28.1)	18.2 (27.2)	0.7 (− 4.7, 6.1)	0.02	16	0	65	0	19
Insomnia	95	35.8 (33.1)	42.5 (33.8)	6.7 (0.9, 12.4)	0.20	16	0	51	0	34
Appetite loss	97	19.6 (26.7)	18.9 (28.8)	− 0.7 (− 7.0, 5.7)	0.03	20	0	62	0	19
Constipation	97	14.8 (28.0)	15.8 (24.6)	1.0 (− 4.2, 6.3)	0.04	20	0	57	0	24
Diarrhoea	97	20.3 (28.3)	18.9 (29.2)	− 1.4 (− 7.8, 5.1)	0.05	27	0	55	0	19
Financial problems	97	16.5 (24.6)	16.5 (26.8)	0.0 (− 4.4, 4.4)	0.00	15	0	69	0	15
EORTC QLQ-GINET21										
Muscle/bone pain symptom	95	23.2 (29.6)	29.1 (33.1)	6.0 (− 0.6, 12.5)	0.20	19	0	48	0	33
Sexual function	51	28.8 (37.7)	33.3 (36.5)	4.6 (− 3.8, 13.0)	0.12	16	0	61	0	24
Information/communication function	93	17.9 (28.9)	10.8 (20.9)	− 7.2 (− 13.5, − 0.9)	0.25	23	0	69	0	9
Body image	95	17.9 (32.5)	14.0 (28.2)	− 3.9 (− 9.9, 2.2)	0.12	0	0	72	0	28
Endocrine scale	98	12.9 (16.2)	12.5 (17.9)	− 0.4 (− 3.5, 2.7)	0.02	24	0	52	0	23
GI NET scale	97	22.6 (18.4)	20.7 (17.9)	− 2.0 (− 5.5, 1.6)	0.11	29	13	24	10	24
Treatment-related symptoms scale	38	15.8 (17.1)	25.1 (24.0)	9.4 (2.4, 16.4)	0.55	27	2	22	7	41
GI NET social functioning scale	96	38.8 (20.9)	33.7 (21.3)	− 5.1 (− 9.4, − 0.7)	0.24	48	0	25	0	27
Disease-related worries scale	97	52.9 (31.1)	47.3 (30.3)	− 5.6 (− 11.2, − 0.1)	0.18	47	3	22	1	27

*M* mean, *SD* standard deviation, *M diff* mean difference (follow-up minus baseline), *CI* confidence interval, *pts* points

^a^For PROMIS, higher scores reflect poorer outcomes. For QLQ-C30 global health status and functioning scales, higher scores reflect better health status and functioning; for all other scales and items, higher scores reflect worse outcomes. For QLQ-GINET21, higher scores reflect worse outcomes

^b^Kazis effect sizes interpreted as per Cohen’s *d*: trivial, < 0.2; small-sized, 0.2–0.49; medium-sized, 0.5–0.79; and large-sized, ≥ 0.8

^c^Data are valid percentages

Apart from differences on the Anxiety and Sleep Disturbance scales, differences between baseline and follow-up PROMIS scale scores were not significant, and 95% confidence intervals included zero. Differences on the Anxiety and Sleep Disturbance scales were small-sized (ES = 0.30 and 0.20, respectively), with 36% of participants reporting an improvement in anxiety and 33% reporting a deterioration in sleep. Greater than 20% of participants reported deterioration on fatigue, and satisfaction with social roles, and activities scales (32% and 22%, respectively). Near equal percentages of patients also reported improvements (19% and 26%, respectively).

Similarly, apart from differences on the insomnia item (ES = 0.20), differences between baseline and follow-up EORTC QLQ-C30 scale/item scores were not significant, and 95% confidence intervals included zero. Approximately, one-third (34%) of participants reported worse insomnia (at least 5 points) at follow-up. For the EORTC QLQ-GINET21, a medium-sized difference was observed on the treatment-related symptoms scale (ES = 0.55, 41% of participants deteriorated). Small-sized differences were observed on the muscle/bone pain symptom item (ES = 0.20, 33% of participants deteriorated), information/communication function item (ES = 0.25, 23% of participants improved), and the social functioning scale (ES = 0.24, 48% of participants improved). Greater than 20% of participants deteriorated across several scales; however, near equal percentages also reported improvements.

The top ten most prevalent supportive care needs at baseline and follow-up (ordered by highest to lowest percentage reporting moderate/high need at baseline) are presented in Table 3. All mapped to the psychological and health system and information needs domains.

**Table 3:** Top 10 most prevalent moderate-to-high supportive care needs at baseline and follow-up (*n* = 100)

Domain	Supportive care need item	Baseline			Follow-up		
		Responses	%moderate or high need	Rank	Responses	%moderate or high need	Rank
Psych	Concerns about the worries of those close to you	98	47	1	99	40	2
Psych	Uncertainty about the future	100	46	2	97	38	4
Psych	Fears about the cancer spreading	98	46	3	96	46	1
HSI	Being informed about things you can do to get yourself well	98	43	4	99	35	6
HSI	Being informed about your test results as soon as feasible	99	41	5	98	34	8
HSI	Having one member of hospital staff with whom you can talk to about all aspects of your condition, treatment and follow-up	96	40	6	98	38	5
HSI	Being informed about cancer which is under control or diminishing (that is, remission)	97	39	7	99	34	7
Psych	Worry that the results of treatment are beyond your control	99	36	8	99	39	3
HSI	Being adequately informed about the benefits and side-effects of treatments before you choose to have them	99	36	9			
Psych	Learning to feel in control of your situation	100	36	10			
HSI	Having access to professional counselling if you/family/friends need it				98	33	9
HSI	Being treated like a person not just another case				98	31	10

*Psych* psychological needs, *HSI*, health system and information needs

### Patient-reported experiences

Descriptive statistics for CPES items at baseline and follow-up are provided in Table 4. Responses to several items at both assessments indicated high proportions of patients with sub-optimal care experiences.

**Table 4:** Descriptive statistics for CPES items for all NET patients at baseline and follow-up time points. *Denotes questions only asked at baseline

Section/item	Baseline		Follow-up	
	*n*	%	*n*	%
Your diagnosis: thinking of your cancer diagnosis				
1. How do you feel about the way you were told you had cancer?*				
More positive	80	80.0		
Less positive	19	19.0		
*Not included*	*0*	*-*		
2. Did you understand the explanation of what is wrong with you?*				
More positive	32	32.0		
Less positive	68	68.0		
*Not included*	*0*	*-*		
3. When you were told about your cancer, were you given written information about the type of cancer you had?*				
More positive	28	30.8		
Less positive	63	69.2		
*Not included*	*9*	*-*		
4. If you received written information about your cancer, how useful was it?				
Very useful	27	32.5	37	37.4
Somewhat useful	25	30.1	29	29.3
Not very useful	1	1.2	3	3.0
Not useful at all	0	0	1	1.0
Didn't receive written information	30	36.1	29	29.3
* Missing*	*17*	*-*	*1*	*-*
5. Overall, how well do you feel you understand your cancer?				
Very well	15	15.6	20	20.0
Well	61	63.5	62	62.0
Not so well	20	20.8	17	17.0
Not well at all	0	0	1	1.0
*Missing*	*4*	*-*	-	-
6. Overall, how well do you feel your doctor understands your cancer?				
Very well	61	63.5	74	75.5
Well	27	28.1	18	18.4
Not so well	6	6.3	4	4.1
Not well at all	2	2.1	2	2.0
*Missing*	*4*	*-*	*2*	*-*
Treatment Options:				
7. Have you had discussions about possible treatment options with your cancer doctor in the past 3 months?				
Yes (go to Q8)	89	89	67	67
No (go to Q9)	10	10	31	31
*Missing*	*1*	*1*	*2*	*2*
8. Were you given the right amount of (written or verbal) information about treatment options? (*n* = 89 at baseline/*n* = 6 7 at follow-up)				
More positive	69	80.2	44	81.5
Less positive	17	19.8	8	14.8
*Not included*	*3*	*-*	*15*	*-*
9. Have you been involved as much as you wanted to be in decisions about your care and treatment?				
More positive	60	61.9	47	56.0
Less positive	37	38.1	37	44.0
*Not included*	*3*	*-*	*16*	*-*
Specialist nurse or care coordinator				
10. Have you been given the name of a Specialist Nurse/Care Coordinator who would be in charge of your care?				
Yes	65	65	52	52
No (go to Q13)	33	33	32	32
*Missing*	*2*	*2*	*16*	*16*
11. How easy is it for you to contact your Specialist Nurse/Care Coordinator? (*n* = 65 at baseline/*n* = 52 at follow-up)				
More positive	35	72.9	30	69.8
Less positive	13	27.1	13	30.2
*Not included*	*17*	*-*	*9*	*-*
12. When you have important questions to ask your Specialist Nurse/Care Coordinator, how often do you get answers you can understand? (*n* = 65/52)				
More positive	39	81.3	34	82.9
Less positive	9	18.8	7	17.1
*Not included*	*17*	*-*	*11*	*-*
Information and support for people with cancer and their carers				
13. Have hospital staff given you information about support or self-help groups for people with cancer? (e.g. Cancer Helpline)				
More positive	57	74.0	48	73.8
Less positive	20	26.0	17	26.2
*Not included*	*23*	*-*	*35*	*-*
14. When you have important questions to ask your cancer doctor, how often do you get answers you can understand?				
More positive	69	71.9	65	71.4
Less positive	27	28.1	26	28.6
*Not included*	*4*	*-*	*9*	*-*
15. Have you seen information (such as leaflets, posters, information screens etc.) about cancer research in your hospital?				
More positive (yes)	60	60.6	60	62.5
Less positive (no)	39	39.4	36	37.5
*Not included*	*1*	*-*	*4*	*-*
16. Are you able to discuss any worries or fears with hospital staff?				
More positive	62	73.8	61	75.3
Less positive	22	26.2	20	24.7
*Not included*	*16*	*-*	*19*	*-*
17. If your family or someone else close to you wants to talk to your cancer doctor, do they have enough opportunity to do so?				
More positive	52	59.8	51	58.0
Less positive	35	40.2	37	42.0
*Not included*	*13*		*12*	
18. Has your cancer doctor or nurse specialist given your family or someone close to you all the information they need to help care for you at home?				
More positive	32	39.0	34	41.0
Less positive	50	61.0	49	59.0
*Not included*	*18*	*-*	*17*	*-*
19. Have you or your family tried to access information about your cancer online?				
More positive	43	53.8	44	58.7
Less positive	37	46.3	31	41.3
*Not included*	*20*	*-*	*25*	*-*
20. If you found information about your cancer online, how useful was it?				
More positive	22	28.6	23	29.1
Less positive	55	71.4	56	70.9
* Not included*	*23*	*-*	*21*	*-*
21. How much information were you given about your condition and treatment?				
More positive	62	66.7	68	73.1
Less positive	31	33.3	25	26.9
*Not included*	*7*	*-*	*7*	-

At follow-up, responses to the following items indicated high percentages of suboptimal experiences of care item 9, 44% of patients indicated a less positive experience in regards to being involved as much as they wanted to be in decisions about their care and treatment; item 17, 42% reported a less positive experience about their family or someone close to them having the opportunity to talk to their cancer doctor; item 18, 59% reported less positive experiences about having their cancer doctor or nurse specialist give their family or someone close to them, all the information they need to help care for them at home; item 19, 41% indicated a less positive experience about either themselves or their family trying to access information about their cancer online; and item 20, 71% indicated a less positive experience about the usefulness of information about NETs online.

## Discussion

The majority of psychosocial outcomes and care experiences of patients with NETs at 6 months follow-up were similar to those reported at baseline (within 6 months of diagnosis); score differences on most scales/items were trivial (i.e. less than small-sized). Some small sized differences were observed; on average, insomnia and sleep disturbance were worse compared to baseline, as were muscle/bone pain symptoms. Treatment-related symptoms and body image were also worse, on average compared to baseline. This is likely attributable to more patients undergoing active treatment at follow-up compared with baseline [28]. Conversely, at follow-up, approximately one-third of patients had at least a 5-point improvement in anxiety. Whilst this was a small-sized difference, scores at both time points for all PROMIS domains did not cross mild-to-moderate thresholds and were within normative standards [29], and therefore, average scores were not considered to be of clinical significance.

Identifying how the outcomes and experiences of NET patients have changed over time is informative given there is evidence to suggest that there is a relationship between QoL and clinical severity which can change over time [30, 31]. However, domains in which a stabilisation or no change is reported at 6-month follow-up are equally important. This can provide valuable insight into the persistent and unique issues patients with NETs face. As previously published in Guccione et al. (2022), compared with general population norms, patients with NETs report significant financial difficulties. Financial toxicity is well reported in the literature [19, 32, 33], and results from this study suggest these are persistent, with 84% of participants reporting ongoing or worsening financial problems compared to baseline.

While there was a small-sized improvement in information/communication function overall (QLQ-GINET21), only 23% of patients exhibited a meaningful improvement based on individual scores. High percentages of patients were still reporting either not receiving written information or for those who are receiving written information, not finding it useful (CPES), 6 months after baseline data collections. From the sample, 57% of NET patients are also still reporting at least one moderate-to-high health system information needs (SCNS-SF). The inadequacy of provision of information and the dominant theme of unmet information needs is a reoccurring one 6 months beyond initial reporting at baseline. Information needs of people with cancer are typically highest during the early stages of their diagnosis [34]. Research has found that across various cancer diagnoses, information needs will also begin to decline beyond 4 months from diagnosis [35]; however, this was observed in only 22% of this sample.

Information needs are persistently a high priority for patients with NETs, most likely due to the low provision of information. Patients with NETs continue to report difficulties in accessing and understanding written information that is persistent over time. There are many benefits of being adequately informed. Improved knowledge has been reported to provide patients with a sense of empowerment and is associated with reducing anxiety [36, 37]. Whilst information from online resources is often reported as preferred source in large proportions of people who are diagnosed with cancer [20], overwhelmingly, 71% report information available online about their cancer not to be useful.

NETs are a rare and heterogeneous cancer; therefore, given the clinical variability between patients, navigating through information resources to find information that is specific and relevant can be complex. The provision of high-quality comprehensible information is essential in providing best cancer care, and yet, results from this study suggest that access to requisite information is a persistent unmet need among people with NETs. People with cancer that are poorly informed are at greatest risk of not engaging with medical decision making, experiencing greater anxiety and are also more likely to seek alternative therapies that lack scientific support [38].

### Study limitations

Eligibility criteria required participants to be able to speak and read English, and therefore, the findings from this study have not included culturally and linguistically diverse populations or those that speak a language other than English. These population in particular often report worse quality of life [39], cancer outcomes, and the greatest problems with accessing information relevant to their cancer in their preferred language [40].

Treatment regimens were not controlled for in this study. Participants were undergoing a range of treatments consisting of chemotherapy, somatostatin analogues, peptide receptor, radio nucleotide Lutate, surgery, radiotherapy, or combinations of treatments. Therefore, different treatments may have had different impacts on the experiences reported by participants.

### Clinical implications

The utility of improved resources and intervention packages is clear from the findings of this study. Unmet information needs are persistent for people with NETs. The development of multimodal informational resources that address the information needs of patients with NETs is essential to improve the best care for these patients.

## Conclusions

The results of this study confirm the need to improve the provision of information for people with NETs. Information relevant to diagnosis and beyond is either not available/inaccessible or does not adequately meet their needs. These findings will inform the design and development of an informational resource that will facilitate patient access and understanding of information on NETs.
